# Disparities in COVID-19 infection, hospitalisation and death in people with schizophrenia, bipolar disorder, and major depressive disorder: a cohort study of the UK Biobank

**DOI:** 10.1038/s41380-021-01344-2

**Published:** 2021-12-07

**Authors:** Lamiece Hassan, Niels Peek, Karina Lovell, Andre F. Carvalho, Marco Solmi, Brendon Stubbs, Joseph Firth

**Affiliations:** 1 Division of Psychology and Mental Health, The University of Manchester, Manchester Academic Health Science Centre, Manchester, M13 9PL UK; 2 Centre for Health Informatics, Division of Informatics, Imaging and Data Sciences, The University of Manchester, Manchester, M13 9PL UK; 3 NIHR Greater Manchester Patient Safety Translational Research Centre, The University of Manchester, Manchester, UK; 4 Division of Nursing, Midwifery and Social Work, The University of Manchester, Manchester Academic Health Science Centre, Manchester, M13 9PL UK; 5 IMPACT (Innovation in Mental and Physical Health and Clinical Treatment) Strategic Research Centre, School of Medicine, Barwon Health, Deakin University, Geelong, VIC Australia; 6 Psychiatry Department, University of Ottawa, Ottawa, ON Canada; 7 The Ottawa Hospital, University of Ottawa, Ottawa, ON Canada; 8 Ottawa Hospital Research Institute (OHRI) Clinical Epidemiology Program, University of Ottawa, Ottawa, ON Canada; 9 Department of Psychological Medicine, Institute of Psychiatry, Psychology, Neuroscience (IoPPN), King’s College London & South London and Maudsley NHS Foundation Trust, London, UK

**Keywords:** Psychiatric disorders, Schizophrenia, Bipolar disorder, Depression

## Abstract

People with severe mental illness (SMI; including schizophrenia/psychosis, bipolar disorder (BD), major depressive disorder (MDD)) experience large disparities in physical health. Emerging evidence suggests this group experiences higher risks of infection and death from COVID-19, although the full extent of these disparities are not yet established. We investigated COVID-19 related infection, hospitalisation and mortality among people with SMI in the UK Biobank (UKB) cohort study. Overall, 447,296 participants from UKB (schizophrenia/psychosis = 1925, BD = 1483 and MDD = 41,448, non-SMI = 402,440) were linked with healthcare and death records. Multivariable logistic regression analysis was used to examine differences in COVID-19 outcomes by diagnosis, controlling for sociodemographic factors and comorbidities. In unadjusted analyses, higher odds of COVID-19 mortality were seen among people with schizophrenia/psychosis (odds ratio [OR] 4.84, 95% confidence interval [CI] 3.00–7.34), BD (OR 3.76, 95% CI 2.00–6.35), and MDD (OR 1.99, 95% CI 1.69–2.33) compared to people with no SMI. Higher odds of infection and hospitalisation were also seen across all SMI groups, particularly among people with schizophrenia/psychosis (OR 1.61, 95% CI 1.32–1.96; OR 3.47, 95% CI 2.47–4.72) and BD (OR 1.48, 95% CI 1.16–1.85; OR 3.31, 95% CI 2.22–4.73). In fully adjusted models, mortality and hospitalisation odds remained significantly higher among all SMI groups, though infection odds remained significantly higher only for MDD. People with schizophrenia/psychosis, BD and MDD have higher risks of COVID-19 infection, hospitalisation and mortality. Only a proportion of these disparities were accounted for by pre-existing demographic characteristics or comorbidities. Vaccination and preventive measures should be prioritised in these particularly vulnerable groups.

## Introduction

The health inequalities for people with severe mental illness (SMI) are regarded as a human rights issue [1, 2]. Specifically, individuals living with depression, schizophrenia/psychosis and other psychoses, or bipolar disorder (BD) - are at much heightened risk of physical diseases, which is the primary cause of the ~15 year gap in life expectancy between people with SMI and the general population [3]. To date, much of the attention on these physical health disparities has focused on the elevated rates of cardiovascular [4] and metabolic diseases in SMI [5, 6], as these account for notably higher proportions of premature mortality observed in this population other than suicide or accidental deaths [3, 7].

The COVID-19 pandemic has highlighted the importance of considering how infectious diseases may disproportionately affect people with SMI. Even before the pandemic, there has been considerable evidence, particularly from low and middle income countries, which has already indicated the heightened morbidity and mortality associated with SMI due to various infectious diseases [8–10]. Alongside this, studies in high-income settings have shown that infectious diseases are more prevalent in people with SMI [11–13], and have been the leading cause of preventable hospitalisations in this population even prior to the pandemic [14]. Until recently, however, this topic has received relatively little attention.

Within the first few months of COVID-19 affecting Western countries, evidence quickly began to emerge to show that people with SMI are disproportionately affected. First, a nationwide case-control study in the United States (US) found that patients with recent diagnoses of mental disorders had higher risks of COVID-19 infection, hospitalisation and death [15]. Since then, a series of studies have since emerged from large-scale studies of electronic health records around the world. This emerging evidence base has been summarised in recently meta analyses: Vai et al. [16] identified 23 studies eligible for meta analysis, while Fond et al. [17] and Toubasi et al. [18] both identified 16. These have confirmed the susceptibility of people with SMI to COVID-19 risks, particularly among people with psychosis.

Though there are mounting concerns about the vulnerability of people with mental illness to COVID-19 [19, 20], the current picture of how SMI is associated with COVID-19 outcomes is incomplete. In particular, rather than considering SMI overall, it is important to clarify how disparities in outcomes differ across the diagnostic spectrum. Whilst studies have consistently confirmed associations between mortality and schizophrenia/psychosis, other relationships are arguably less clear. For instance, while Vai et al. [16] found that the risk of hospitalisation was increased among people with any mental disorder, after stratifying by diagnosis psychotic disorders were not significantly associated. Furthermore, they found no studies that distinguished between unipolar and bipolar mood disorders.

Several studies have also identified a varying range of pertinent clinical, demographic and lifestyle factors which may attenuate the risk of COVID-19 related outcomes among people with SMI. In particular, several of the physical diseases identified as risk factors for COVID-19 are known to be more prevalent among people with SMI, highlighting the inherent complexity in disentangling causal relationships [21–23].

Separately to this, generating local data is crucial in the context of COVID-19 pandemic, since countries have been differently affected by COVID-19 and have dealt with the virus differently. Indeed, country had a significant effect on mortality estimates in Vai’ et al.’s meta-analysis [16]. Understanding around how COVID-19 has affected those with SMI living in the UK (a country which has seen particularly high rates of COVID-19 infections and deaths across the entire population) has to date been limited to a handful of studies, focused on the first wave of COVID-19 infections [24–27]. In particular, Yang et al. [27] used the UK Biobank (UKB) cohort to explore relationships with a range of clinically confirmed psychiatric disorders, reporting in particular the elevated risk of COVID-19 related hospitalisations and mortality.

Therefore, in this study, we aim to build upon and update the findings of previous studies in the UK and internationally, by further establishing the risk of COVID-19-related infection, hospitalisation and mortality in people with SMI compared to the general UK population, stratified by diagnosis. We further aim to account for the sociodemographic, clinical and behavioural factors which may attenuate risks of COVID-19 infection, hospitalisation and deaths among people with SMI. In doing this, the study aims to confirm and contribute to the existing literature in this emergent field, to better understand the disproportionate risks faced by people living with SMI, and to inform mental health professionals and health policy makers in the UK.

## Methods

### Design and participants

We used anonymised data from UKB, which captures health information and linked health records for half a million men and women enroled in a prospective cohort study between 2006 and 2010 [28]. At baseline UKB participants were aged 40–69 years of age and resided in England, Scotland or Wales within 10 miles of a UKB assessment centre. On enrolment, they participated in detailed physical assessments and structured interviews to provide information about their current and previous lifestyle behaviours, as well as physical and mental health.

Data about hospital episodes and associated diagnoses are linked with UKB via England’s Hospital Episode Statistics (HES) database, the Scottish Morbidity Record and the Patient Episode Database for Wales. NHS Digital and Scotland’s NHS Central Register provide continuously updated information about deaths. For the purposes of COVID-19 related research, linkage has also been enabled with primary care records, providing additional information about primary care diagnoses and prescriptions. Data about any available COVID-19 test results were provided by Public Health England (PHE) and Public Health Scotland (PHS).

All UKB participants who were alive on 31.01.2020 (the first COVID-19 related death in the UK), attended their first UKB assessment in England or Scotland, and had not withdrawn consent were eligible for inclusion in the current study. Using the latest UKB data releases for HES, PHE and PHS data, participants were followed up until 28th February 2021. As up-to-date HES data for Wales was not available at the time, we excluded people who attended any of the assessment centres in Wales as a methodological precaution.

All UKB participants gave written consent on enrolment for their data to be used by approved researchers and are free to withdraw their data at any time. UKB has approval from the North West Multi-centre Research Ethics Committee.

### Exposures and outcomes

We searched UKB’s database and linked electronic health records (EHRs)– including HES records, primary care records and UKB’s algorithmically defined diagnosis variables - for evidence of SMI (lifetime) up until 31.01.2020. Full lists of the ICD-10, SNOMED-CT and Read codes used to define SMI and other diagnoses in this study can be found in Supplementary Table 1. For the purposes of this study, we assigned each participant to one of four hierarchically defined, diagnostic groups: schizophrenia/psychosis; bipolar disorder (excluding schizophrenia/psychosis); major depressive disorder (MDD, excluding schizophrenia/psychosis or bipolar disorder); or no evidence of SMI.

The primary outcome was risk of COVID-19 related mortality, defined as all reported deaths that identified COVID-19 (either confirmed or suspected i.e. ICD codes U07.1 or U07.2) as the underlying cause of death. Secondary outcomes included risk of lab-confirmed COVID-19 infections (any record of a positive polymerase chain reaction test result vs. a negative result or no record of a test) and hospitalisations due to COVID-19 (any HES record of an inpatient stay accompanied by ICD diagnosis codes U07.1 or U07.2 vs. none).

We ascertained three separate, binary COVID-19 outcomes for each participant within the study observational period, which were hierarchically defined: died due to COVID-19; hospitalised due to COVID-19 (including those who died due to COVID-19); infected (including those who died or were hospitalised due to COVID-19); or no evidence of COVID-19. It is possible some individuals with COVID-19 died without being hospitalised (e.g. care home residents) or, due to the evolving nature of testing capacity and policy in the UK, ever having tested positive, or even being tested for, COVID-19. Nonetheless, we made pragmatic assumptions as our intention was to compare death, hospitalisation and infection as markers of relative severity of COVID-19 outcomes.

We also explored how demographic and clinical factors affected risk of COVID-19 outcomes among people with and without SMI. Demographic data included age (in years), sex and ethnicity (White; mixed; Asian; Black; Chinese; other). Townsend deprivation index scores were provided by UKB as a measure of material deprivation, calculated based on residential postcode data recorded at participants’ initial baseline assessment and accompanying national census data. Individuals’ scores across the dataset were divided into equal quintiles; quintile 1 representing the least deprived and quintile 5 representing the most deprived. We also included comorbidity data (UKB recorded diagnoses up until 31.01.2020) for the following conditions, suspected to be associated with COVID-19 [29] and available in UKB with a sufficiently large sample size (>1000 cases): asthma, cancer, chronic kidney disease, chronic obstructive pulmonary disease (COPD), coronary heart disease (CHD), diabetes, liver disease, neurological disorders (including epilepsy Parkinson’s disease and dementia) and rheumatoid arthritis. Smoking status (ever; never) and Body Mass Index (BMI), as recorded at baseline assessment were also used.

### Statistical analysis

We investigated the impact of SMI on mortality and hospitalisation due to COVID-19, adjusted for demographic and clinical factors. All statistical analyses were conducted using R version 3.6.2. In preparation for analysis, UKB routinely perform cleaning and data validation checks on externally collected data to increase the accuracy of records linkage and exclude invalid codes. We found missing demographic and/or lifestyle data from that provided at initial baseline assessments among 6827 of the participants eligible for inclusion in the study (*N* = 454,123). We compared sample characteristics for the whole cohort and complete cases (*N* = 447,296), the latter excluding participants with missing data (Supplementary Table 2). As we found similar results and the overall proportion of cases excluded was small (<2%), we have opted to report only the results of the complete case analysis here.

First, descriptive statistics (percentages and counts) were used to determine the prevalence of COVID-19 outcomes among the sample, by diagnostic group. These were arranged in a flow diagram, illustrating the proportion of individuals experiencing increasingly severe outcomes within diagnostic subgroups. Then, to estimate risks for each SMI group, we used multivariable logistic regression analysis to generate crude and adjusted odds ratios (ORs) with 95% confidence intervals, using people with no evidence of SMI as the reference group. Three model variations were used: crude (model 1); demographically adjusted (model 2), adjusted for age, ethnicity, sex and deprivation; and fully adjusted (model 3), adjusted for demographic variables plus physical comorbid diagnoses, BMI and smoking history. This allowed us to interpret absolute risks to people with SMI (model 1), while iteratively eliminating the effects of potential confounders and/or mediators (models 2 and 3) to understand the role of demographic and physical comorbidities in attenuating risks.

Diagnosis was entered into models as an unordered factor. Statistical tests were conducted with significance set at *p* < 0.05 (two-sided).

In adjusted models, sex, comorbid diagnoses and smoking status were included as binary variables. Deprivation was entered as a categorical variable, while age and BMI were entered as continuous variables. Due to the over-representation of White ethnicities in UKB (with White ethnicities representing 95% of total sample size), ethnicity was recoded and included in models as a binary variable with people from all other backgrounds—henceforth referred to as ethnic minority groups—pooled together (White vs ethnic minorities). While sub-optimal, this was necessary to generate a sufficient sample size to enable any kind of ethnic comparison.

## Results

Baseline demographic and clinical characteristics of the final sample are described in Table 1 (*N* = 447,296). Over half (55%) of the sample was female and the mean age was 67 years. The vast majority of the sample identified as White (95%). Overall, 0.4% (*n* = 1925) of the sample had evidence of a diagnosis of schizophrenia/psychosis or psychosis, 0.3% (*n* = 1483) had BD and 9% (*n* = 41,448) had MDD. The remainder (90%; *n* = 402,440) had no evidence of SMI recorded in their records. The most commonly recorded physical health problems were cancer (11%), followed by CHD (8%), asthma (7%) and diabetes (5%).

**Table 1:** Baseline sample characteristics (as of 31.01.2020, unless otherwise indicated).

	Schizophrenia/Psychosis		BD		MDD		None (ref)	
	*n*	%	*n*	%	*n*	%	*n*	%
Sex^1^								
Female	942	48.9	900	60.7	27,797	67.1	217,969	54.2
Male	983	51.1	583	39.3	13,651	32.9	184,471	45.8
Age group								
Mean, SD	65.5	8.4	66.5	8.1	66.5	8.1	67.3	8.1
Ethnicity^1^								
White	1660	86.2	1412	95.2	39,442	95.2	380,032	94.4
Mixed	39	2.0	10	0.7	325	0.8	2306	0.6
Asian	61	3.2	22	1.5	675	1.6	8226	2.0
Black	115	6.0	22	1.5	575	1.4	6763	1.7
Other	50	2.6	17	1.1	431	1.0	5113	1.2
Deprivation quintile^1^								
1 (−6.26, −3.94)	159	8.3	209	14.1	6808	16.4	82,761	20.6
2 (−3.94, −2.78)	204	10.6	246	16.6	7281	17.6	82,117	20.4
3 (−2.78, −1.31)	234	12.2	274	18.5	7852	18.9	81,273	20.2
4 (−1.31, 1.32)	387	20.1	273	18.4	8644	20.9	80,068	19.9
5 (1.32, 11)	941	48.9	481	32.4	10,863	26.2	76,221	18.9
BMI^1^								
Mean, SD	28.4	5.6	28.7	5.7	28.3	5.5	27.2	4.6
Smoking status^1^								
Never	667	34.6	498	33.6	14,851	35.8	166,641	41.4
Ever	1258	65.4	985	66.4	26,597	64.2	235,799	58.6
History of physical illness								
Asthma	224	11.6	207	14.0	5726	13.8	25,642	6.4
Cancer	208	10.8	160	10.8	5149	12.4	360,123	10.5
CKD	65	3.4	83	5.6	1089	2.6	5047	1.3
COPD	142	7.4	99	6.7	2159	5.2	7310	1.8
CHD	239	12.4	189	12.7	5373	13.0	32,135	8.0
Diabetes	259	13.5	167	11.3	3912	9.4	20,081	5.0
Liver disease	66	3.4	56	3.8	1166	2.8	4262	1.1
Neurological	264	13.7	193	13.0	4307	10.4	15,874	3.9
Rheumatoid arthritis	40	2.1	42	2.8	1034	2.5	4344	1.1
TOTAL	1925	100	1483	100	41,448	100	402,440	100

^1^As recorded at initial UKB assessment.

**Fig. 1:**
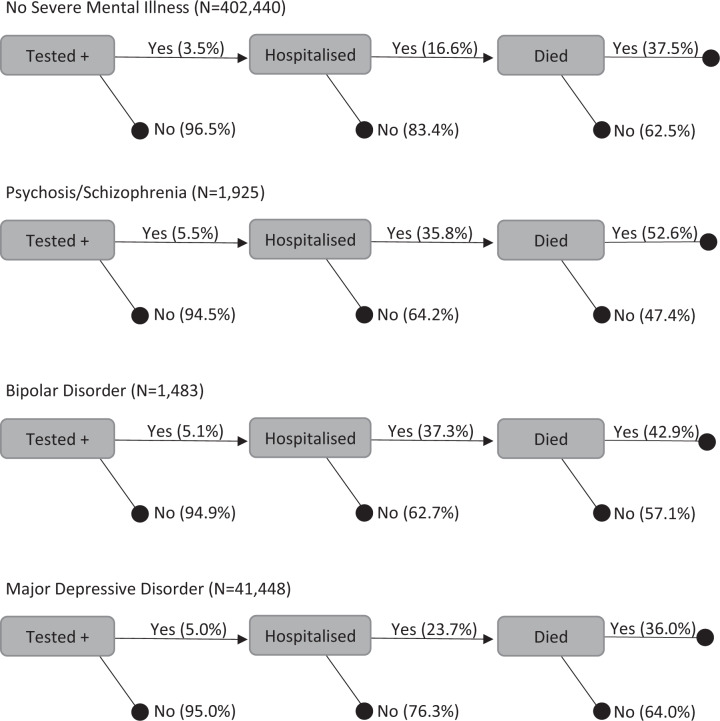
COVID-19 outcomes, by psychiatric diagnosis. Increasingly severe COVID-19 outcomes are shown as relative proportions (%) among those with different diagnoses known to have been infected, hospitalised and/or died. As outcomes were hierarchically defined (see methods), it is possible some individuals with COVID-19 died without being hospitalised or ever having tested positive for COVID-19. Pragmatic assumptions were made to compare death, hospitalisation and infection as markers of relative severity of COVID-19 outcomes.

A total of 16,282 people tested positive for COVID-19, while 2885 were hospitalised and 1081 died (Tables 2–4). Fig. 1 shows the proportion of individuals among those known to have contracted COVID-19 who experienced hospitalisation and/or death as a result. Among people with COVID-19, those with schizophrenia/psychosis and BD had the highest rates of infection leading to hospitalisation; 35.8% and 37.3%, respectively, compared to 16.6% among with those without SMI. Among people who were hospitalised due to COVID-19, over half of those with schizophrenia/psychosis (52.6%) did not survive.

**Table 2:** Odds of infection due to COVID-19, by psychiatric diagnosis.

		Unadjusted	Adjusted^1^ (demographic)	Fully adjusted^2^ (demographic + clinical)
Diagnosis	*n* (%)	0 R (95% CI)	aOR (95% CI)	aOR (95% CI)
None (ref. group)	14,011 (3.5)	1	1	1
MDD	2090 (5.0)	1.47 (1.40–1.54)^a^	1.42 (1.36–1.49)^a^	1.24 (1.18–1.30)^a^
Bipolar	75 (5.1)	1.48 (1.16–1.85)^a^	1.39 (1.09–1.74)^a^	1.15 (0.90–1.44)
Schizophrenia	106 (5.5)	1.61 (1.32–1.96)^a^	1.29 (1.05–1.56)^a^	1.10 (0.90–1.34)
All	16,282			

^1^Adjusted for age, ethnicity, sex and deprivation.

^2^Adjusted for demographic variables plus asthma, cancer, chronic kidney disease, chronic obstructive pulmonary disease, coronary heart disease, diabetes, liver disease, neurological disorders, rheumatoid arthritis, BMI and smoking history.

^a^Indicates *p* < 0.05.

**Table 3:** Odds of hospitalisation due to COVID-19, by psychiatric diagnosis.

		Unadjusted	Adjusted^1^ (demographic)	Fully adjusted^2^ (demographic + clinical)
Diagnosis	*n* (%)	OR (95% CI)	aOR (95% CI)	aOR (95% CI)
None (ref. group)	2324 (0.6)	1	1	1
MDD	495 (1.2)	2.08 (1.89–2.29)^a^	2.28 (2.06–2.51)^a^	1.65 (1.48–1.82)^a^
Bipolar	28 (1.9)	3.31 (2.22–4.73)^a^	3.28 (2.20–4.70)^a^	2.20 (1.46–3.18)^a^
Schizophrenia/psychosis	38 (2.0)	3.47 (2.47–4.72)^a^	2.85 (2.02–3.90)^a^	2.12 (1.50–2.90)^a^
All	2885			

^1^Adjusted for age, ethnicity, sex and deprivation.

^2^Adjusted for demographic variables plus asthma, cancer, chronic kidney disease, chronic obstructive pulmonary disease, coronary heart disease, diabetes, liver disease, neurological disorders, rheumatoid arthritis, BMI and smoking history.

^a^Indicates *p* < 0.05.

**Table 4:** Odds of mortality due to COVID-19, by psychiatric diagnosis.

		Unadjusted	Adjusted^1^ (demographic)	Fully adjusted^2^ (demographic + clinical)
Diagnosis	*n* (%)	OR (95% CI)	aOR (95% CI)	aOR (95% CI)
None (ref. group)	871 (0.2)	1	1	1
MDD	178 (0.4)	1.99 (1.69–2.33)^a^	2.29 (1.94–2.69)^a^	1.62 (1.37–1.91)^a^
Bipolar	12 (0.8)	3.76 (2.00–6.35)^a^	3.86 (2.05–6.55)^a^	2.49 (1.30–4.29)^a^
Schizophrenia/psychosis	20 (1.0)	4.84 (3.00–7.34)^a^	4.21 (2.60–6.43)^a^	3.14 (1.92–4.81)^a^

^1^Adjusted for age, ethnicity, sex and deprivation.

^2^ Adjusted for demographic variables plus asthma, cancer, chronic kidney disease, chronic obstructive pulmonary disease, coronary heart disease, diabetes, liver disease, neurological disorders, rheumatoid arthritis, BMI and smoking history.

^a^Indicates *p* < 0.05.

Table 2 shows the ORs for COVID-19 infection associated with different SMI diagnoses. When compared against people with no evidence of SMI, unadjusted models showed significant associations between COVID-19 infection and schizophrenia/psychosis (OR = 1.61, 95% CI 1.32–1.96), BD (OR = 1.48, 95% CI 1.16–1.85) and MDD (OR = 1.47, 95% CI 1.40–1.54). After adjustment for demographic and clinical factors, only the association with MDD (OR = 1.24 95% CI 1.18–1.30) remained statistically significant.

When considering odds of hospitalisations due to COVID-19, the crude model yielded an OR of 3.47 (95% CI 2.47–4.72) among people with a history of schizophrenia/psychosis, 3.31 (95% CI 2.22–4.73) among people with BD and 2.08 (95% CI 1.89–2.29) among people with MDD (Table 3). The odds of hospitalisation remained significantly elevated among all three SMI groups even after adjustment for demographic and clinical factors, approximately doubling the odds in people with a history of schizophrenia/psychosis or BD.

Table 4 shows the ORs for the primary outcome of COVID-19 mortality associated with different SMI diagnoses. When compared against people with no evidence of SMI, unadjusted models showed an OR of 4.84 (95% CI 3.00–7.34) for mortality among people with a history of schizophrenia/psychosis, 3.76 (95% CI 2.00–6.55) for people with BD and 1.99 (95% CI 1.69–2.33) among people with MDD. Again, after adjustment for demographic and clinical factors, all three SMI groups remained at significantly higher risk for mortality, with the highest risk among those with schizophrenia/psychosis (OR = 3.14, 95% CI 1.92–4.81).

Table 5 reports the ORs for individual variables included in the fully adjusted regression models. This indicates that schizophrenia/psychosis ranked highest in terms of the strength of association with  mortality, over all other demographic and clinical variables, and second highest with hospitalisation (second only to BD). Age, ethnic minority status, male gender, BMI, smoking status, CHD, CKD, COPD, diabetes, liver disease, neurological problems and rheumatoid arthritis were consistently associated with increased risks of COVID-19 related infection, hospitalisation and mortality.

**Table 5:** Fully adjusted^1^ multivariable model for odds of mortality, hospitalisation and infection due to COVID-19, by diagnosis.

Variable	Infection aOR (95% CI)	Hospitalisation aOR (95% CI)	Mortality aOR (95% CI)
Diagnosis			
None	1	1	1
MDD	1.24 (1.18–1.30)^a^	1.65 (1.48–1.82)^a^	1.62 (1.37–1.91)^a^
Bipolar	1.15 (0.90–1.44)	2.20 (1.46–3.18)^a^	2.49 (1.30–4.29)^a^
Schizophrenia/psychosis	1.10 (0.90–1.34)	2.12 (1.50–2.90)^a^	3.14 (1.92–4.81)^a^
Age	0.96 (0.96–0.96)^a^	1.07 (1.06–1.07)^a^	1.13 (1.12–1.14)^a^
White vs. ethnic minorities	1.50 (1.42–1.58)^a^	1.77 (1.54–2.02)^a^	1.93 (1.54–2.40)^a^
Female vs Male	1.10 (1.07–1.14)^a^	1.75 (1.62–1.89)^a^	1.94 (1.71–2.21)^a^
Deprivation			
Q1 vs. Q2	1.08 (1.02–1.14)^a^	1.13 (0.99–1.30)	1.11 (0.89–1.39)
Q1 vs. Q3	1.19 (1.13–1.25)^a^	1.28 (1.12–1.47)^a^	1.26 (1.01–1.57)^a^
Q1 vs. Q4	1.24 (1.18–1.30)^a^	1.42 (1.25–1.62)^a^	1.44 (1.16–1.78)^a^
Q1 vs. Q5	1.41 (1.34–1.48)^a^	2.12 (1.87–2.40)^a^	2.17 (1.78–2.66)^a^
BMI	1.03 (1.03–1.03)^a^	1.05 (1.05–1.06)^a^	1.07 (1.05–1.08)^a^
Never vs. ever smoked	1.03 (1.00–1.05)^a^	1.08 (1.02–1.14)^a^	1.15 (1.05–1.27)^a^
Comorbidity			
Asthma	1.12 (1.06–1.19)^a^	1.24 (1.10–1.39)^a^	0.96 (0.79–1.17)
Cancer	1.05 (0.99–1.11)	1.20 (1.08–1.32)^a^	1.12 (0.97–1.33)
CHD	1.18 (1.12–1.25)^a^	1.30 (1.17–1.43)^a^	1.21 (1.04–1.40)^a^
CKD	1.48 (1.32–1.65)^a^	1.80 (1.53–2.10)^a^	1.72 (1.35–2.16)^a^
COPD	1.50 (1.37–1.64)^a^	1.84 (1.60–2.11)^a^	1.70 (1.36–2.10)^a^
Diabetes	1.34 (1.26–1.42)^a^	1.55 (1.39–1.72)^a^	1.74 (1.48–2.05)^a^
Liver disease	1.14 (1.01–1.28)^a^	1.59 (1.30–1.92)^a^	1.78 (1.31–2.37)^a^
Neurological	1.37 (1.29–1.46)^a^	1.94 (1.73–2.16)^a^	2.22 (1.87–2.61)^a^
Rheumatoid arthritis	1.27 (1.12–1.44)^a^	1.62 (1.30–1.99)^a^	1.88 (1.36–2.54)^a^

^1^Adjusted for demographic variables plus asthma, cancer, chronic kidney disease, chronic obstructive pulmonary disease, coronary heart disease, diabetes, liver disease, neurological disorders, rheumatoid arthritis, BMI and smoking history.

^a^Indicates *p* < 0.05.

Comparison between ORs yielded by the demographically adjusted and fully adjusted models (i.e model 2 vs. model 3) allowed us to estimate the percent excess risk of mortality explained by clinical variables. On this basis, pre-existing comorbidities explained 33.3% of the excess risk for people with schizophrenia/psychosis ((4.21–3.14)/(4.21–1)*100), 47.9% for people with BD ((3.86–2.49)/(3.86–1)*100) and 51.9% for people with MDD ((2.29–1.62)/(2.29–1)*100).

## Discussion

This study has investigated COVID-19 related disparities for people with SMI in the UKB, and the pertinent factors which may explain these relationships. Our findings confirm those of several, large-scale studies internationally which have reported increased risks of COVID-19 infections, hospitalisations and mortality among people with mental illnesses [15, 21, 22, 30, 31]. Specifically, there was a five-fold increase in the odds of mortality and a three-fold increase for odds of hospitalisation among people with schizophrenia/psychosis. Our findings advance on previous findings using UKB [24–27] to examine data from both the first and second waves of COVID infections and stratifying outcomes among people with different psychiatric diagnoses.

The increased odds observed were partly explained by differences in demographic characteristics and pre-existing physical comorbidity. Previous studies have linked male sex, belonging to an ethnic minority, older age and certain respiratory and metabolic conditions to higher rates of adverse COVID-19 outcomes [15, 32, 33]. Our findings concur with prior evidence that these established sociodemographic and clinical factors contribute to increased risks seen among people with psychiatric diagnoses; yet they do not appear to fully explain them. In fully adjusted models, pre-existing comorbidities explained 33.3–51.9% of the excess mortality risk among people with SMI once demographic factors had been accounted for. This suggests alternative pathways not measured by this study may have been at work. For example, emerging literature points towards the importance of biological processes, including biomarkers of inflammation—already known to be linked with psychiatric disorders [34]—in explaining disease severity among people with COVID-19 [35].

A strength of this paper is the large sample afforded from the UKB, providing sociodemographic, lifestyle, and health-related data from a substantial portion of the UK population. Indeed, this study is one of a handful internationally to stratify a range of COVID-19 outcomes by psychiatric diagnoses, rather than pooling SMI, including distinguishing people with bipolar disorder, an under-researched clinical group in this context [16]. Nonetheless, there are several different ways of defining psychiatric diagnoses in UKB. In this study we only examined lifetime SMI. It is possible that this definition may have masked differential risks between those with historical illnesses and those with more recent diagnoses. Furthermore, diagnoses were based on coded EHR data and did not include self-reported illnesses, thus it is possible some diagnoses were missed.

A limitation of this study is the general homogeneity among the UKB participants, who are older (mostly over 65 years of age) and less ethnically diverse than the general population (95% White). As with other volunteer-based cohort studies, there is evidence of a ‘healthy volunteer’ bias in UKB [36], which means the sample cannot be regarded as representative. Selection bias and/or collider bias may have affected our results [37]. In particular, SMI rates among UKB participants were lower than in the UK population generally [38]; even among the SMI population, it is likely that a healthier subset of individuals were unintentionally recruited.

Our findings relating to infections using UKB should be interpreted with caution as testing was non-random and thus at risk of collider bias [37]. Changes in testing capacity and strategy during the first and second waves of coronavirus in the UK (initially testing was focused in hospitals and among healthcare workers) mean that rates of infection have likely been underestimated. Due to test eligibility restrictions, people with asymptomatic infections may not have been tested. Furthermore, our statistical approach did not account for deaths due to other causes, which also may have resulted in underestimations of risks. While we opted for logistic regression to generate ORs, aiding comparability with several previous major studies [e.g. 15, 21, 23], alternative approaches may have better dealt with censoring issues.

Collectively, this means that our findings should not be extrapolated to estimate the true prevalence of COVID-19 related infections, hospitalisations and mortality in the UK population. Nonetheless, the strength, direction and consistency of the associations observed across the various analyses applied, and in the context of findings from previous studies, does increase confidence in our principal finding that individuals with SMI are at substantially increased risk of serious COVID-19 related outcomes, particularly hospitalisation and death, compared to the general population.

The implications of our study are twofold. First, with regards to practical implications, our study further supports recent calls to public health officials [19, 20] to go beyond age and physical morbidity based targeting and urgently prioritise people with SMI as an extremely high-risk group, to attenuate the disparities and prevent serious outcomes resulting from COVID-19 infection. Such approaches could include prioritising access to vaccination programs for people with SMI, and/or increasing the rates of screening and testing for COVID-19 (and other infectious diseases). Second, with regards to future research, there is a need for studies with more representative samples of the UK population to further explore and delineate the risk factors underpinning increased risks among particular subgroups of people within SMI groups. These could include exploration of the role of psychotropic prescribing in COVID-19 outcomes, which has been attracting interest [39, 40]. In addition, given the notably higher rates of cardiovascular and metabolic diseases observed in the SMI sample, future research on this topic should further examine the extent to which elevated risks in SMI samples may be mediated by individuals having multiple comorbid conditions. Consideration could be given to statistical approaches which allow better decomposition of the direct and indirect effects of SMI to disentangle such pathways.

In conclusion, this is the most comprehensive study to date to examine COVID-19 outcomes in people with different SMI diagnoses in the UK population. The rich data afforded by the UKB allowed for investigation of the contributing sociodemographic, clinical and lifestyle related risk factors for COVID-19 infections, hospitalisations and deaths among different diagnostic subgroups of people with SMI. Results showed that people with SMI, and particularly those with schizophrenia/psychosis, experience significantly higher odds of infection, hospitalisation and death from COVID-19, which cannot be fully accounted for by sociodemographic, clinical or lifestyle predisposing factors. From this, there is now a need for future research to better understand the driving factors and biological mechanisms behind the observed relations, along with an urgency to develop and evaluate strategies for attenuating these risks these vulnerable populations.

## Supplementary information

Supplemental Material
