# Supplementary material for: Disparities in COVID-19 infection, hospitalisation and death in people with schizophrenia, bipolar disorder, and major depressive disorder: a cohort study of the UK Biobank
Source: Mol Psychiatry. 2021 Dec 7;27(2):1248–55. doi: 10.1038/s41380-021-01344-2 (PMC9054655; doi:10.1038/s41380-021-01344-2)
Supplement: Supplementary file 1 — Supplemental Material [file 41380_2021_1344_MOESM1_ESM.docx]

**SUPPLEMENTARY INFORMATION**

**Supplementary Table 1:** Code lists for documented SMI and physical health diagnoses

| **Diagnosis** | **Data source (UKB item, if applicable)** | **Coding system** | **Codes** | **Description** |
| --- | --- | --- | --- | --- |
| Schizophrenia / psychosis | UKB - Diagnoses (41202/41204) | ICD-10 | F20-29 | Schizophrenia/psychosis, schizotypal and delusional disorders |
|  | EMIS | SNOMED | 69322001  58214004 | Psychotic disorder  Schizophrenia/psychosis |
|  | TPP | Read | X00S6  X00Qx  E10*  Eu2*  XE1aM | Psychotic disorder  Psychotic episode NOS  Schizophrenic disorders  Schizophrenia/psychosis, schizotypal and delusional disorders  Schizophrenic psychoses (& [paranoid schizophrenia/psychosis]) |
| Bipolar | UKB - Diagnoses (41202/41204) | ICD-10 | F31* | Bipolar affective disorder |
|  | EMIS | SNOMED | 13746004 | Bipolar affective disorder |
|  | TPP | Read | X00SM  Eu31*  E117*  XaB95  XE1ZX | Bipolar affective disorder  Bipolar affective disorder  Unspecified bipolar affective disorder  Other manic-depressive psychosis  Bipolar affective disorder |
| MDD | UKB - Diagnoses (41202/41204) | ICD-10 | F32  F33 | Major depressive disorder, single episode  Major depressive disorder, recurrent |
|  | EMIS | SNOMED | 36923009  832007 | Major depression, single episode  Moderate major depression |
|  | TPP | Read | XSEGJ  XE1Y0  XE1Y1  E112*  E113* | Major depressive disorder  Single major depressive episode  Recurrent major depressive episodes  Depression: [single major episode] or [agitated] or [endogenous (including first episode)]  Recurrent depression: [major episode] or [endogenous] |
| Asthma | UKB - Diagnoses (41202/41204) | ICD-10 | J45 | Asthma |
| Cancer | UKB - Diagnoses (41202/41204) | ICD-10 | C* | Neoplasms |
| CHD | UKB - Diagnoses (41202/41204) | ICD-10 | I2* | Ischemic heart diseases, pulmonary heart disease and diseases of pulmonary circulation |
| CKD | UKB - Diagnoses (41202/41204) | ICD-10 | N18* | Chronic renal failure |
| COPD | UKB - Diagnoses (41202/41204) | ICD-10 | J43*  J44* | Chronic obstructive pulmonary disease  Other chronic obstructive pulmonary disease |
| Diabetes | UKB - Diagnoses (41202/41204) | ICD-10 | E10-E14 | Diabetes mellitus |
| Liver disease | UKB - Diagnoses (41202/41204) | ICD-10 | K70-K77 | Diseases of liver |
| Neurological conditions | UKB - Diagnoses (41202/41204) | ICD-10 | G12*  G40*  G8*  G20*  G30*  F00-F03 | Spinal muscular atrophy and related syndromes (inc motor neurone disease)  Epilepsy  Cerebral palsy and other paralytic syndromes  Parkinson disease  Alzheimer disease  Dementia in Alzheimer disease, vascular dementia, dementia in other diseases classified elsewhere and unspecified dementia. |
| Rheumatoid arthritis | UKB - Diagnoses (41202/41204 | ICD-10 | M05  M06 | Seropositive rheumatoid arthritis  Other rheumatoid arthritis |

**Supplementary Table 2:** Sample characteristics– comparison of complete cases versus whole cohort

|  | Whole cohort | | Complete cases | |
| --- | --- | --- | --- | --- |
| Variable | N | % | N | % |
| Sex |  |  |  |  |
| Female | 250,988 | 55.3 | 247,608 | 55.4 |
| Male | 203,134 | 44.7 | 199,688 | 44.6 |
| Missing | 1 | <0.01 | - | - |
| Age group (mean, SD) | 67.22 | 8.11 | 67.22 | 8.10 |
| Missing | 1 | <0.01 | - | - |
| Ethnicity |  |  |  |  |
| White | 426,119 | 93.8 | 422,546 | 94.5 |
| Mixed | 2,717 | 0.6 | 2,680 | 0.6 |
| Asian | 9,337 | 2.1 | 8,984 | 2.0 |
| Black | 7,692 | 1.7 | 7,475 | 1.7 |
| Chinese | 1,484 | 0.3 | 1,460 | 0.3 |
| Other | 4,279 | 0.9 | 4,151 | 0.9 |
| Missing | 2,495 | 0.5 | - | - |
| Deprivation score (mean, SD) | -1.31 | 3.08 | -1.33 | 3.08 |
| Missing | 568 | 0.1 | - | - |
| BMI (mean, SD) | 27.36 | 4.75 | 27.35 | 4.75 |
| Missing | 2,674 | 0.6 | - | - |
| Smoking status |  |  |  |  |
| Never | 184,482 | 40.6 | 182,657 | 40.8 |
| Ever | 267,082 | 58.8 | 264,639 | 59.2 |
| Missing | 2,559 | 0.6 | - | - |
| TOTAL | 454,123 | 100 | 447,296 | 100 |
